# Ultra-sensitive detection of protein biomarkers for diagnosis of Alzheimer’s disease†
†Electronic supplementary information (ESI) available. See DOI: 10.1039/c6sc05615f


**DOI:** 10.1039/c6sc05615f

**Published:** 2017-03-24

**Authors:** Hei-Nga Chan, Di Xu, See-Lok Ho, Man Shing Wong, Hung-Wing Li

**Affiliations:** a Department of Chemistry , Hong Kong Baptist University , Hong Kong , China . Email: mswong@hkbu.edu.hk ; Email: hwli@hkbu.edu.hk

## Abstract

Beta amyloid peptide, tau, and phosphorylated tau are well recognized as promising biomarkers for the diagnosis of Alzheimer’s disease (AD). In this work, we developed a direct, versatile, and ultrasensitive multiplex assay for the quantification of trace amounts of these protein biomarkers for AD in different types of biological fluids including cerebrospinal fluid, serum, saliva, and urine. The detection assay is based on the immunoreaction between the target proteins and their corresponding pair of antibodies followed by fluorescence labelling with a newly developed indolium-based turn-on fluorophore, namely **SIM**. **SIM** was tailor-made as a reporter to provide a high signal-to-noise ratio for the detection assay. An exceptionally low limit of detection down to the femto-molar level was achieved in this assay with minute consumption of the sample. This versatile detection assay is capable of reliably quantifying not only the target proteins simultaneously from a CSF sample in an hour but also trace amounts of protein biomarkers in saliva and urine. This assay has a high potential to serve as a practical tool for the diagnosis of AD.

## Abstract 2

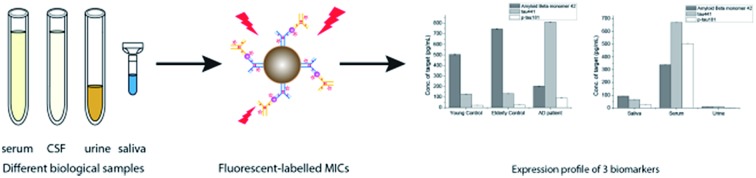
Beta amyloid peptide, tau, and phosphorylated tau are well recognized as promising biomarkers for the diagnosis of Alzheimer’s disease (AD).

## Introduction

In recent decades, Alzheimer’s disease (AD) has drawn tremendous attention from the public as it has become the most common type of dementia, and therefore poses a huge burden to society and the economy. There are over 37 million patients currently suffering from AD and the number is expected to increase 3-fold by 2050.^1^ To date there is no cure for AD, but an early diagnosis and treatment can ameliorate the symptoms of this devastating disease. As reported in the literature, treatment of patients with cholinesterase inhibitors (such as donepezil, rivastigmine, and galantamine) at the early stage not only can significantly preserve the volume of the hippocampus, but also conserve the functional and structural integrity of neurons.^2^

Currently, clinical evaluation, cognitive tests, and neuroimaging are the standard procedures for the complete diagnosis of AD. Cognitive tests, including the Mini-Mental State Examination (MMSE) and the Montreal Cognitive Assessment (MoCA), are able to distinguish mild cognitive impairment from aging by assessing brain functions.^3^,^4^ Neuroimaging is an objective, evidence-based approach for AD diagnosis through monitoring the structural changes of the brain. Positron emission tomography (PET), single photon emission computed tomography (SPECT), and magnetic resonance imaging (MRI) are the most common types of neuroimaging.^5^ Unfortunately, the major drawback of PET and SPECT is the need to administer radioactive imaging agents into a patient. Although MRI is non-invasive, the analysis of the subtle structural and functional abnormalities in these neurodegenerative brains is quite expensive. More importantly, the development of the disease starts long before any noticeable decrease in hippocampal volume or deposition of plaques and tau tangles. Thus, development of a non-invasive assessment of the risk of AD is critical not only for early diagnosis but also for developing therapeutic strategies for AD treatment.

Recently, 42-amino-acid formed from the amyloid-β precursor protein (Aβ_42_) and total (t-tau) and phosphorylated tau (p-tau_181_) proteins were found to be important pathological hallmarks for AD. Researchers discovered that the content of these proteins in the cerebrospinal fluid (CSF) is highly correlated with Alzheimer’s disease occurrence. Most importantly, the changes of the levels of Aβ, tau, and p-tau due to AD occur almost 10–15 years before any symptoms, such as cognitive impairment and decline, are shown.^6^ Monitoring the subtle changes of these protein biomarkers can provide a promising preclinical diagnosis of AD.

To date, there is no sensitive and cost-effective method for the quantification of the aforementioned proteins. Capillary electrophoresis,^7^–^9^ resonance light scattering,^10^ surface plasmon resonance,^11^–^13^ enzyme-linked immunosorbent assay (ELISA),^14^,^15^ and the polymerase chain reaction (PCR)^16^ are the common approaches for peptide detection. However, these methods are limited by the requirements of sophisticated equipment, complicated steps and long assay time, and low sensitivity. To achieve a higher sensitivity, single-molecule detection technologies such as Simoa and Erenna were developed, in which the antibodies are pre-labelled for signalling and an additional chemical reaction is required to break down the immunocomplexes for detection.

Herein, we report a direct, rapid, inexpensive, versatile, and ultrasensitive detection assay for the simultaneous quantification of the Aβ_42_, tau and p-tau_181_ proteins in different biological fluids including cerebrospinal fluid, saliva, serum, and urine. The cost of the newly developed assay is 1–2 orders of magnitude lower than that of the commercially available ELISA kit. Thus, population wide screening and monitoring can be more feasible, which particularly imposes a lower financial burden on developing countries. The detection assay employs magnetic nanoparticles as the purification and preconcentration platform. The target analytes are captured by the antibody immobilized on the nanoparticles and labelled by a newly developed turn-on fluorescent dye, **SIM**. The fluorescent labelled magnetic immunocomplexes are then detected using a fluorescence imaging system. A remarkably low detection limit at the femto-molar level was achieved with minute consumption of the sample (less than 20 µL of the biological fluid for the three AD protein biomarkers) and the results are further validated using INNOTEST ELISA kits. This detection assay can also be performed using a commercial spectrofluorimeter and thus is applicable in a general laboratory setting. It has a high potential for clinical diagnosis.

## Results and discussion

### Physical and binding properties of the fluorophores

**Fig. 1:**
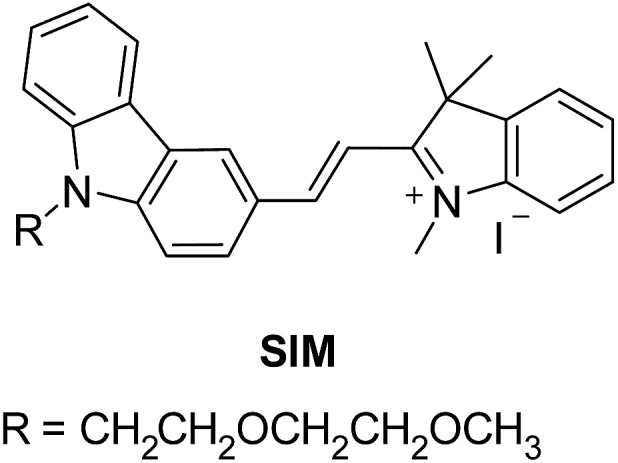
Molecular structure of **SIM**.

The chemical structure and the synthetic scheme of the newly developed fluorophore for Aβ_42_, tau, and phosphorylated-tau detection is shown in Fig. 1 and Scheme S1,† respectively. We previously reported a fluorophore, namely **SLAce**, for the fluorescence labelling of cancer protein biomarkers. However, we found that **SLAce** does not bind strongly with the smaller AD biomarkers to give rise to a large turn-on fluorescence signal for sensitive analysis. Instead, we have developed a novel indolium-based turn-on fluorophore, namely **SIM**, which affords a strong fluorescence enhancement upon binding with the AD biomarkers. **SIM** also showed superior photophysical properties including fluorescence enhancement when compared with the commercial dyes *i.e.* thioflavin T, thioflavin S, Congo red, and phycoerythrin for use in detecting Aβ_42_ and tau. Hence, the sensitivity and the cost-effectiveness of the detection assay can be significantly improved. As listed in Table S1,† both **SIM** and **SLAce** fluorophores can be excited by a 488 nm laser but exhibit two very different emission maxima (597 and 681 nm, respectively) which would facilitate multiplex detection. Furthermore, **SIM** was also found to be photostable, and its fluorescence was salt-independent and was not affected by the presence of the nanoparticles (Fig. S1–S3†).

### Detection scheme

**Fig. 2:**
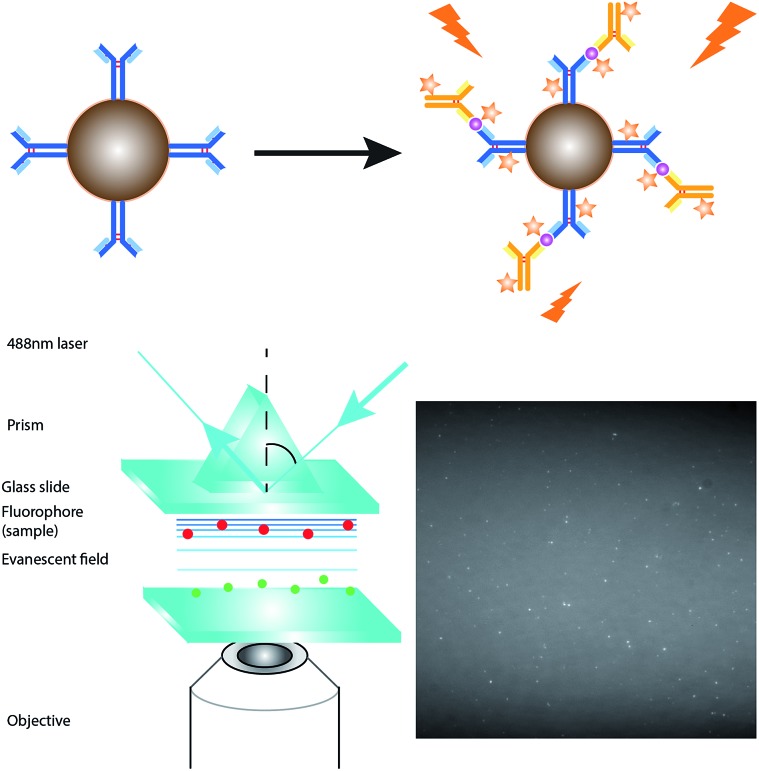
Schematic illustration of the detection assay for the direct quantification of target protein biomarkers.

Fig. 2 depicts the detection scheme of the developed detection assay. The nanoprobes were prepared by conjugating the APTES activated silica-coated iron oxide nanoparticles with the monoclonal primary capture antibody for Aβ_42_ (clone 12F4, epitope C-terminus), tau_441_ (clone BT2, epitope aa 194–198), and p-tau_181_ (clone AT270, epitope pT181)^17^–^21^ with the aid of a crosslinker, glutaraldehyde (GA). The silica-coated iron oxide nanoparticles were prepared as previously reported. In short, the iron oxide nanoparticles were prepared by co-precipitation and coated with silica using a sol–gel method. The resulting nanoparticles were characterised using TEM and the average diameter of the particles is 100 ± 15 nm (Fig. S4†). The magnetic nanoprobes were incubated with the biomarkers and detection antibody sequentially. The magnetic immunocomplexes were then labelled with the fluorescent dye and injected into a flow cell fabricated from two cover slides. An external magnetic field was provided by placing a magnet on the cover slides to attract the nanoprobes towards the cover slide/solution interface for TIRFM imaging.

When compared with a surface-based reaction, such as in the SPR measurement, which is performed on the glass slide, the magnetic nanoparticles provide a larger reaction surface and a higher efficiency, and thus a higher signal-to-noise ratio. The solution-based reaction provides a higher efficiency for the immunoreaction since both the nanoprobes and target analytes freely diffuse in solution. Besides, the immunocomplexes are formed on the surface of the magnetic nanoparticles. Not only are the targets pre-concentrated, but also the magnetic target immunocomplexes can be moved away from the unwanted reactants to the online detection zone simply using a small magnetic bar.

### Optimization of the immunoassay

The sensitivity of the detection assay is crucial for the analysis of the protein biomarkers that have low abundances in the CSF and other biological fluids. To maximize the detection efficiency and the sensitivity of the assay, a series of optimization tests were conducted. Firstly, to ensure the maximum coverage of the capture antibody on the nanoparticles, the nanoprobes were prepared by incubating 1 nM, 500 pM, and 100 pM concentrations of the capture antibody with the same concentration of both the target protein and detection antibody. The higher the fluorescence intensity, the higher the coverage of the capture antibodies able to capture the target. As shown in Fig. S5A,† the nanoparticles were saturated with the capture antibody when the nanoparticles were immobilized with 1 nM of the capture antibody. Hence, the capture antibody concentration of 1 nM was selected for the detection assay. An optimal concentration of the nanoprobes not only prevents the aggregation of the magnetic immunocomplexes (found in ≥600 µg mL^–1^ nanoprobes), but also provides sufficient reaction platforms for capturing the protein targets. Fig. S5B† demonstrates that a concentration of 30 µg mL^–1^ of the nanoprobes is insufficient but 300 µg mL^–1^ of the nanoprobes provides a higher fluoresence intensity for 10 pM of the target. Hence, a concentration of 300 µg mL^–1^ of nanoprobes was used for the detection. In general, the fluorescence signal obtained increases with the applied concentration of the fluorophore. The immunocomplexes were incubated with 1, 10, 20, 50, 100, and 200 µM of the turn-on fluorophore. As illustrated in Fig. S5C,† the intensity increased sharply when the dye concentration went up from 1 µM to 50 µM, reached a maximum at 50 µM, and levelled off when the concentration increased beyond 50 µM. This is because, beyond 50 µM, a higher amount of dye contributes to higher background noise. Thus, 50 µM of fluorescent dye was the optimum dye concentration for the detection assay. The incubation procedure also affects the antigen–antibody interaction. In that regard, as there is a thousand-fold difference in size between the monomeric Aβ_42_ and the detection antibody, the steric hindrance is large for the nanoprobes approaching the target in the target-detection antibody complexes in the right orientation. Consequently, the target has a smaller chance of being approached by the capture antibody and so fewer targets can be captured. As depicted in Fig. S5D,† the resulting signal was higher when the nanoprobes were incubated with the target and detection antibody sequentially, as compared with when it was performed simultaneously. Thus, the detection assay was performed in 2 steps. Lastly, the optimal time for the immunoreaction was determined by measuring the average net intensity of the magnetic immunocomplexes after 15, 30, or 60 min of incubation for each step. Fig. S5E and F† demonstrate that the resulting intensity increased with the incubation time and reached a maximum at 30 min, which was set as the reaction time for the detection assay.

### Direct quantification of the biomarkers using a single particle measurement

In order to demonstrate the performance of the developed detection assay, a calibration plot of the average net intensity as a function of the target concentration was established under the optimal conditions. Different concentrations of Aβ_42_, tau_441_, and p-tau_181_ (0–1000 fM) were incubated with the probes and detection antibodies in a 10% artificial CSF matrix and labelled with the **SIM** fluorophore. The limits of detection of Aβ_42_, tau_441_, and p-tau_181_ are 23, 14, and 34 fM, respectively, with good coefficients of determination (*R*^2^ = 0.9993, 0.9988, and 0.9999, respectively). As compared with that using our previously reported fluorophore, **SLAce**, the fluorescence enhancement of **SIM** increased significantly (by more than 6 times) which provides a higher signal-to-noise ratio improving the sensitivity of the detection assay (Fig. S6†). Thus, the LOD for the corresponding target protein is lowered by 50% (Fig. S7A–F†). With regard to the cut-off values of Aβ_42_, t-tau, and p-tau_181_, which are 530 pg mL^–1^ (117.4 pM), 350 pg mL^–1^ (7.6 pM), and 80 pg mL^–1^ (1.7 pM), respectively, the detection assay is capable of quantifying the AD protein biomarkers directly and thus distinguishing AD patients from the population.

### Specificity of the assay

**Fig. 3:**
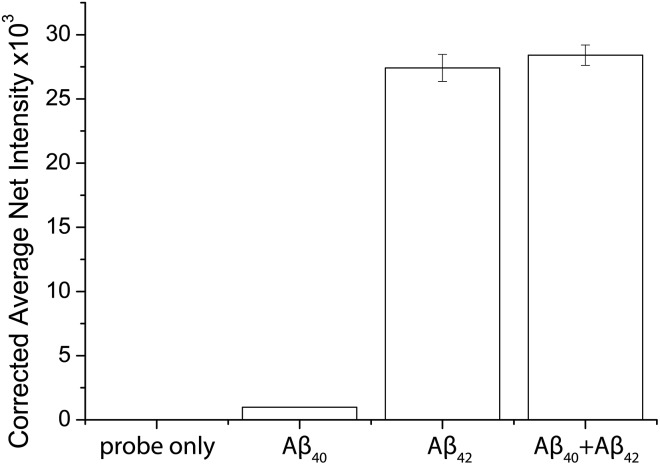
Selectivity of the detection assay. The Aβ_42_ nanoprobes were incubated with 0 fM beta amyloid proteins, 500 fM Aβ_40_, 500 fM Aβ_42_, and a mixture of 500 fM Aβ_40_ and 500 fM Aβ_42_. Error bars show the standard error of mean *n* = 3 (corrected average net intensity = average net intensity of the sample – average net intensity of the probe) (average net intensity = (1 × 1 square pixel of 100 individual MICs) – (1 × 1 square pixel of 100 individual background areas on the image)/100).

The specificity of the probes affects the accuracy of the detection assay significantly. To evaluate the specificity of the assay, the probes for Aβ_42_ were incubated with the target proteins, its homologous protein Aβ_40_, and a mixture of them under optimal conditions. The average net intensities of Aβ_40_ and the mixture of Aβ_40_ and Aβ_42_ increase by 2.4% and 1.4%, respectively (Fig. 3) ((signal from sample – blank)/blank × 100% or (signal from mixtures – signal from target)/signal from target × 100%). There is a negligibly small increase of the signal in the presence of the homologous proteins, implying that the antibody-based probe is capable of distinguishing these two similar peptides which are only different by 2 amino acids. The result agreed with our findings from the specificity study on the unconjugated antibody (Fig. S8†) and literature studies, which reported that the 12F4 antibody is specific to Aβ_42_ due to the fact that the capture antibody (clone 12F4) binds to the C-terminus of the beta-amyloid proteins and is specific for the isoform ending at the 42^nd^ amino acid.^21^

### Quantification of the target proteins in human biological samples

**Fig. 4:**
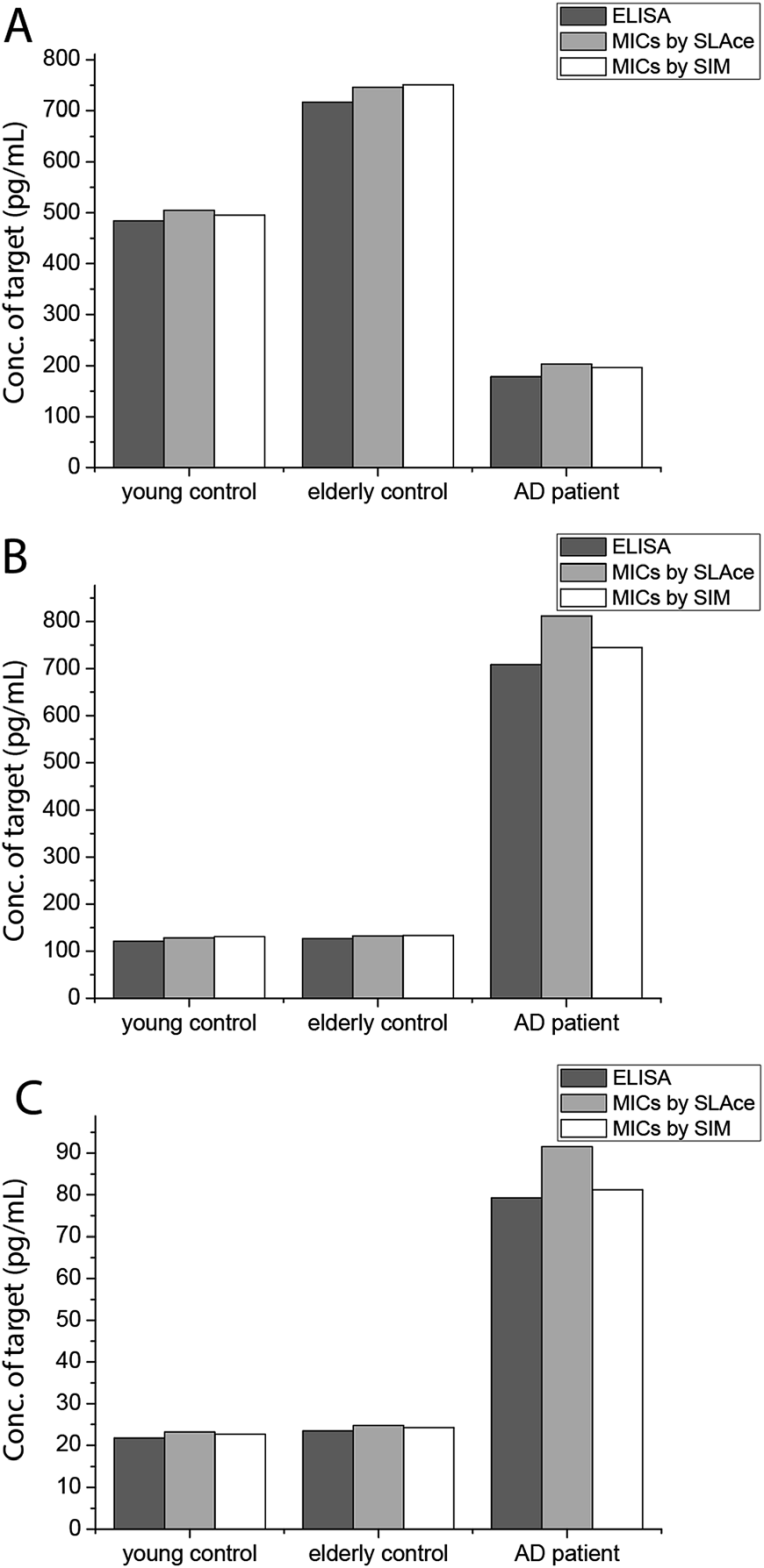
Quantification of (A) Aβ_42_, (B) tau_441_, and (C) p-tau_181_ using ELISA and the developed assay using **SLAce** and **SIM** as the reporters.

**Fig. 5:**
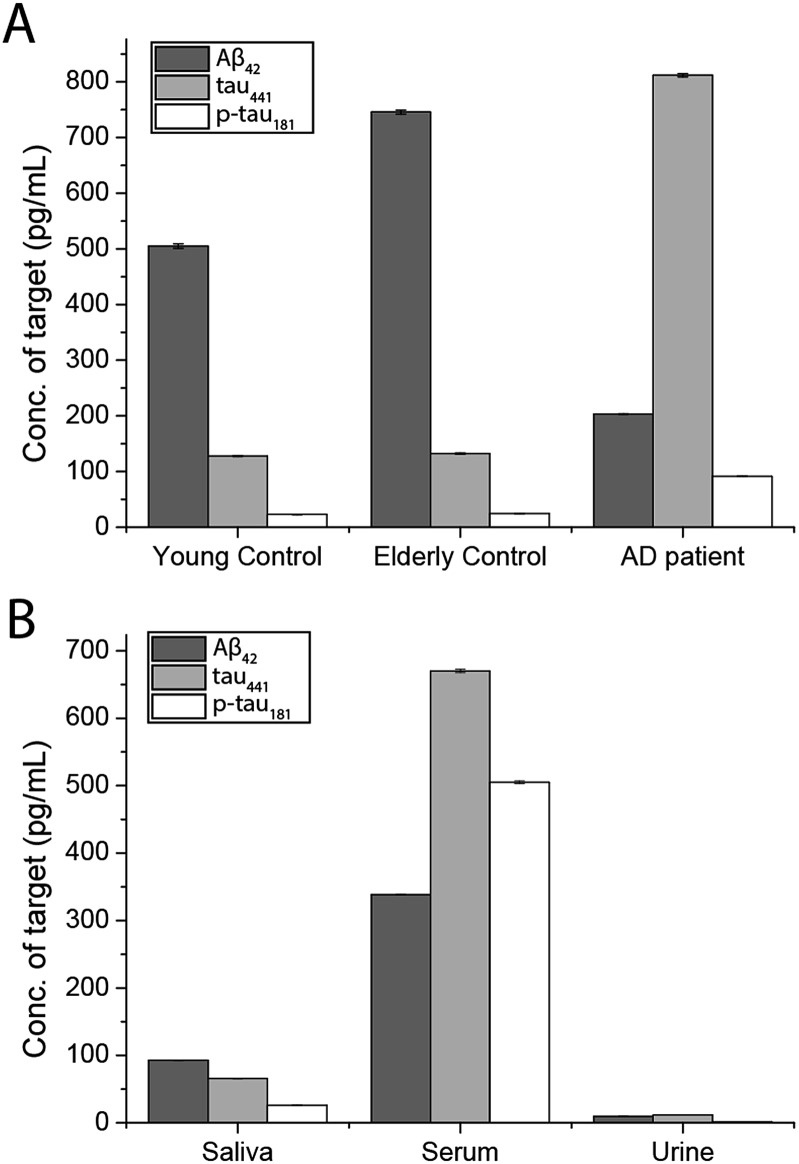
Quantification of the biomarkers in human (A) CSF and (B) different biological samples by external calibration. Error bars show the standard error of mean *n* = 3 (average net intensity = (1 × 1 square pixel of 100 individual MICs) – (1 × 1 square pixel of 100 individual background areas on the image)/100).

To demonstrate the feasibility and potential of the assay for clinical application, we here applied the developed assay to the quantification of the biomarkers in different bodily fluids, including CSF, serum, urine, and saliva. CSF is believed to be the most representative of biochemical changes as it is in direct contact with the extracellular region of the brain. We thus applied the developed assay to quantify the content of biomarkers in crude CSF samples of a healthy young donor (patient # 7515), a healthy old donor (patient # 7577), and an AD patient (patient # 8014). To examine the precision of the assay, the CSF sample was diluted with different dilution factors, ranging from 100- to 5000-fold, for the biomarkers. As shown in Fig. S9,† there is a linear decline of the signal when the sample was diluted from 100- to 5000-fold with a correlation coefficient (*R*^2^) of 0.9949, suggesting the precision of the detection assay is not affected by sample dilution. The content of the Aβ_42_ protein in the patient # 7515, 7577, and 8014 samples is 484.05, 716.73, and 178.67 pg mL^–1^, respectively. The content of the tau_441_ protein in the patient # 7515, 7577, and 8014 samples is 121.52, 126.76, and 708.4 pg mL^–1^, respectively, while that of the p-tau_441_ protein is 21.76, 23.48, and 79.25 pg mL^–1^, respectively.^22^–^26^ The measured results were further validated using the commercially available INNOTEST ELISA kits as shown in Fig. 4, while the profile of the biomarkers is summarized in Fig. 5. This elementary observation supports the previous study showing that the level of Aβ_42_ decreases significantly during the development of AD. We found that the expression profiles of Aβ_42_, tau, and p-tau in CSF for the young control and elderly control were similar. However, there was a very different profile observed in the AD patient’s CSF sample. The assay that we developed should facilitate future massive clinical studies.

Since CSF samples can only be collected by lumbar puncture, it is an invasive procedure that should be performed by trained physicians. To look for non-invasive alternatives, researchers have also explored the content of the target proteins in other biological samples including serum, saliva, and urine for correlation with AD. Nonetheless, the number of studies and the scale of studies have been limited by the availability and sensitivity of the existing testing kits. There are very few literature studies reporting the salivary content of Aβ_42_, tau, and p-tau in AD patients. Zhang *et al.* identified salivary tau and p-tau but not Aβ species, using mass spectrometry.^27^ The Carro group found a small increase in the salivary Aβ_42_ content of AD patients.^28^ Both groups reported that the expression profile of the three biomarkers in saliva and urine correlated well with the progression of AD. However, the abundance of these proteins is much lower than in CSF and serum, making the detection more challenging. Meanwhile, a larger scale study on the potential utility of the salivary Aβ_42_, tau, and p-tau proteins as AD biomarkers is essential for clinical statistical relevance. There is an urgent need for an accurate, fast, highly sensitive, and cost effective detection kit for population-wide AD screening.

To demonstrate the versatility of the detection assay, the levels of the three biomarkers in serum, urine, and saliva were also determined. We first quantified the content of Aβ_42_, tau_441_, and p-tau_181_ in a healthy donor’s serum sample and it was determined to be 340.07, 669.44, and 493.79 pg mL^–1^, respectively. We compared the results obtained by an external calibration method and a standard addition method to investigate the matrix effect on the detection assay. The results depicted in Fig. S10† suggest that they were comparable to each other. Hence, with the usage of the magnetic particles and the specific antibodies, the matrix effect on the detection is not significant. The quantification of the protein biomarkers in a serum sample could be conducted with external calibration.

In view of the fact that the developed assay for the AD protein biomarkers has a detection limit at the femto-molar level, we then determined the levels of salivary and urinary Aβ_42_, tau_441_, and p-tau_181_ in a healthy donor’s sample. The concentrations of the three biomarkers in saliva and urine are much lower than those in CSF and serum. The content of Aβ_42_, tau_441_, and p-tau_181_ in saliva samples of 4 healthy donors was validated with each other at concentrations of around 100, 300, and 30 pg mL^–1^ as shown in Fig. S11,† while those in urine samples were 96.76, 118.68, and 16.88 pg mL^–1^, respectively. These results were consistent with those from the ELISA kits as listed in Table S2.† Both the salivary and urinary content of these three AD proteins was found to be significantly lower than that found in the serum sample (Fig. 5), highlighting the importance of an ultra-sensitive but cost-effective detection assay for population-wide diagnosis of AD.

### Direct quantification of the biomarkers using a commercial fluorimeter

**Fig. 6:**
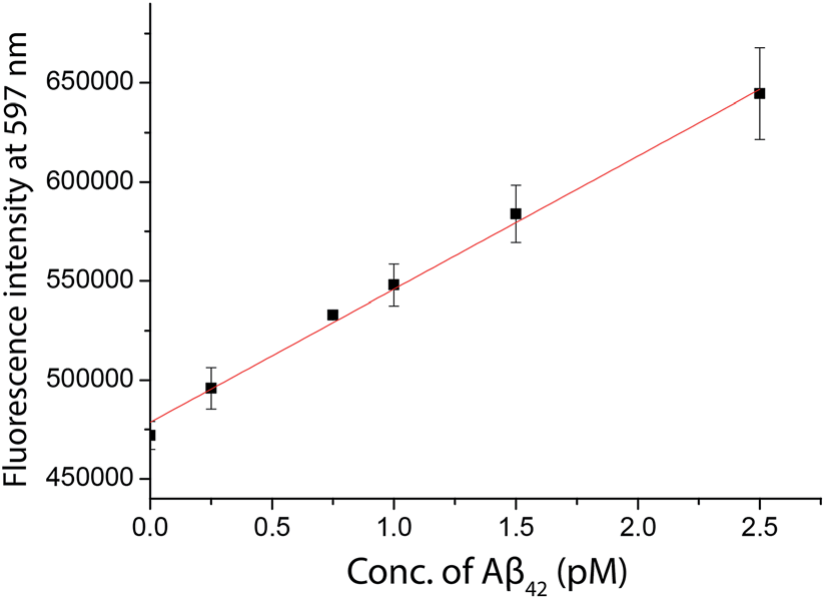
The quantification of Aβ_42_ in human CSF samples as labelled with **SIM** in the presence of a 10% glycerol solution measured by a spectrofluorimeter. A linear range of 0–2500 fM of Aβ_42_ was obtained.

As inspired by the low detection limit achieved, we also investigated the feasibility of coupling the detection approach with a typical fluorimeter. In order to further enhance the fluorescence signal given by the fluorophore in solution, a 10% glycerol solution was added into the final solution. Using **SIM** as the turn-on fluorophore, a linear response, with the limit of detection for Aβ_42_ of 250 fM, was achieved using a conventional spectrofluorimeter (Fig. 6), suggesting that the detection assay was capable of quantifying the biomarkers in a general laboratory setting. As summarized in Table 1, the results obtained by ELISA, TIRFM and the fluorimeter agreed with each other very well. However, the sensitivity afforded by the fluorimeter measurement was found to be inadequate for the detection of tau and p-tau.

**Table 1:** Concentration of Aβ_42_ determined by ELISA, TIRFM, and fluorimeter. (RPD (%) = difference obtained by the developed method and ELISA/concentration obtained by ELISA × 100%)

Sample	ELISA (pM)	TIRFM (pM)	RPD (%)	Fluorimeter (pM)	RPD (%)
Young control	107.21	111.87	4.35	110.28	2.86
Elderly control	158.75	165.17	4.05	155.17	–2.25
AD patient	39.57	37.60	–4.98	39.72	0.36

### Simultaneous detection of Aβ_42_ and tau_441_ in CSF samples

**Fig. 7:**
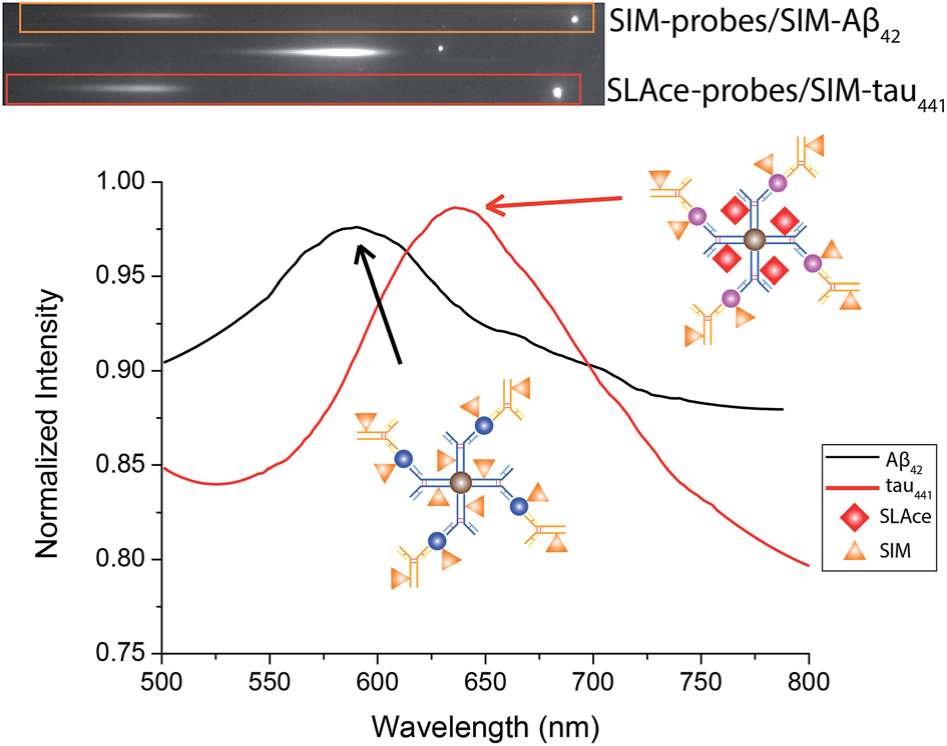
The zero and first order images of the fluorescent labelled magnetic immunocomplexes (top), and the spectra of the magnetic immunocomplexes (bottom).

Multiplex detection improves the accuracy and throughput of an analysis and, most importantly, it reduces the sample consumption and saves time. Taking advantage of the two fluorophores **SLAce** and **SIM**, which could both be excited by a 488 nm laser but possess distinguishable emission profiles, we further demonstrate the capability of the assay for simultaneous quantification of Aβ_42_ and tau_441_ with a minor modification of the established protocol. The antibody-conjugated magnetic probes for Aβ_42_ and tau_441_ were first labelled with **SIM** and **SLAce**, respectively, before the immunoreaction with the target and detection antibodies. The dye-labelled probes were then simultaneously added into the real sample to capture their corresponding target and detection antibodies. The magnetic immunocomplexes formed were then labelled with **SIM**. As a consequence, it gave a target mixture of **SIM**-probes-**SIM**-labelled-Aβ_42_ and **SLAce**-probes-**SIM**-tau_441_. The sample solution was then imaged and analysed using a TIRFM-EMCCD imaging system with a transmission grating. Both the zeroth and first-order fluorescence images were obtained. As shown in Fig. 7, the **SIM**-probes-**SIM**-labelled-Aβ_42_ and **SLAce**-probes-**SIM**-tau_441_ mixtures exhibited emission peaks at 590 nm and 640 nm, respectively. The individual MICs can be readily distinguished and identified using the spectra obtained from the first order images. The quantification of the target can be executed by measuring the zero-order intensity of each MIC.

Calibration curves for the quantification of Aβ_42_ and tau_441_ were constructed (Fig. S12†) for the analysis of real samples. The concentrations of Aβ_42_ in the young control, elderly control and AD patient were determined to be 496.36, 734.39, and 189.7 pg mL^–1^, respectively; while those of tau_441_ were 125.12, 135.61, and 740.96 pg mL^–1^, respectively by this two-color approach, which agreed well with the results obtained from both the ELISA kit (484.05, 716.73, and 178.67 pg mL^–1^ for Aβ_42_ and 121.52, 126.76, and 708.40 pg mL^–1^ for tau_441_) and the singlet detection method (495.42, 750.86, and 196.48 pg mL^–1^ for Aβ_42_ and 131.01, 133.44, and 744.64 pg mL^–1^ for tau_441_).

## Conclusions

In summary, a direct, versatile, and highly sensitive detection assay for the diagnosis of Alzheimer’s disease was developed. With the use of the turn-on indolium-based fluorophore **SIM** and TIRFM, the detection assay can achieve an exceptionally low LOD down to the femto-molar level. The newly developed assay is able to quantify the biomarkers in different biological fluids with minute sample consumption. The results obtained agreed very well with those obtained from the commercially available ELISA kit. The simultaneous detection of Aβ_42_ and tau_441_ can be achieved using a single excitation laser. Other protein biomarkers of interest could be investigated and identified simply by replacing the pair of capture and detection antibodies. This newly developed assay would be practically useful as a low-cost but accurate diagnostic and prognostic tool for AD. It can also serve as a novel alterative non-invasive tool for population-wide screening for AD.

## Supplementary Material

Supplementary information

## References

[cit1] Kaushik A., Jayant R. D., Tiwari S., Vashist A., Nair M. (2016). Biosens. Bioelectron..

[cit2] Alzheimer’s A. (2016). Alzheimer’s Dementia.

[cit3] Ercoli L. M., Siddarth P., Dunkin J. J., Bramen J., Small G. W. (2003). J. Geriatr. Psychiatry Neurol..

[cit4] Conti S., Bonazzi S., Laiacona M., Masina M., Coralli M. V. (2015). Neurol. Sci..

[cit5] Ferreira L. K., Busatto G. F. (2011). Clinics.

[cit6] Blennow K., Dubois B., Fagan A. M., Lewczuk P., de Leon M. J., Hampel H. (2015). Alzheimer’s Dementia.

[cit7] Gonzalez-Dominguez R., Garcia A., Garcia-Barrera T., Barbas C., Gomez-Ariza J. L. (2014). Electrophoresis.

[cit8] Verpillot R., Otto M., Klafki H., Taverna M. (2008). J. Chromatogr. A.

[cit9] Wang Y. R., Yang Y. H., Lu C. Y., Chen S. H. (2015). Anal. Chim. Acta.

[cit10] Yu L., Zhang Y. T., Chen R., Zhang D. H., Wei X. H., Chen F., Wang J. X., Xu M. T. (2015). Talanta.

[cit11] Palladino P., Aura A. M., Spoto G. (2016). Anal. Bioanal. Chem..

[cit12] Vestergaard M., Kerman K., Kim D. K., Hiep H. M., Tamiya E. (2008). Talanta.

[cit13] Wittenberg N. J., Wootla B., Jordan L. R., Denic A., Warrington A. E., Oh S. H., Rodriguez M. (2014). Expert Rev. Neurother..

[cit14] Babic M., Vogrinc Z., Diana A., Klepac N., Borovecki F., Hof P. R., Simic G. (2013). Transl. Neurosci..

[cit15] Wang L. S., Leung Y. Y., Chang S. K., Leight S., Knapik-Czajka M., Baek Y., Shaw L. M., Lee V. M. Y., Trojanowski J. Q., Clark C. M. (2012). J. Alzheimer’s Disease.

[cit16] Kiko T., Nakagawa K., Tsuduki T., Furukawa K., Arai H., Miyazawa T. (2014). J. Alzheimer’s Disease.

[cit17] Xia N., Liu L., Harrington M. G., Wang J. X., Zhou F. M. (2010). Anal. Chem..

[cit18] Portelius E., Westman-Brinkmalm A., Zetterberg H., Blennow K. (2006). J. Proteome Res..

[cit19] Nutu M., Zetterberg H., Londos E., Minthon L., Nagga K., Blennow K., Hansson O., Ohrfelt A. (2013). Dementia Geriatr. Cognit. Disord..

[cit20] Meredith J. E., Sankaranarayanan S., Guss V., Lanzetti A. J., Berisha F., Neely R. J., Slemmon J. R., Portelius E., Zetterberg H., Blennow K., Soares H., Ahlijanian M., Albright C. F. (2013). PLoS One.

[cit21] Gagni P., Sola L., Cretich M., Chiari M. (2013). Biosens. Bioelectron..

[cit22] Petersen R. C., Trojanowski J. Q. (2009). JAMA, J. Am. Med. Assoc..

[cit23] Mattsson N., Zetterberg H., Hansson O. (2009). JAMA, J. Am. Med. Assoc..

[cit24] Buchhave P., Minthon L., Zetterberg H., Wallin K. A., Blennow K., Hansson O. (2012). Arch. Gen. Psychiatry.

[cit25] Tapiola T., Alafuzoff I., Herukka S. K., Parkkinen L., Hartikainen P., Soininen H., Pirttila T. (2009). Arch. Neurol..

[cit26] Andreasen N., Sjogren M., Blennow K. (2003). World J. Biol. Psychiatry.

[cit27] Shi M., Sui Y. T., Peskind E. R., Li G., Hwang H., Devic I., Ginghina C., Edgar J. S., Pan C., Goodlett D. R., Furay A. R., Gonzalez-Cuyar L. F., Zhang J. (2011). J. Alzheimer's Dis..

[cit28] Bermejo-Pareja F., Antequera D., Vargas T., Molina J. A., Carro E. (2010). BMC Neurol..

