# Supplementary material for: Ultra-sensitive detection of protein biomarkers for diagnosis of Alzheimer’s disease
Source: Chem Sci. 2017 Mar 24;8(5):4012–8. doi: 10.1039/c6sc05615f (PMC6094176; doi:10.1039/c6sc05615f)
Supplement: Supplementary file 1 [file SC-008-C6SC05615F-s001.pdf]

Electronic Supplementary Material (ESI) for Chemical Science.
This journal is © The Royal Society of Chemistry 2017

Ultra-sensitive Detection of Protein Biomarkers for Diagnosis of Alzheimer's Disease

Hei-Nga Chan, Di Xu, See-Lok Ho, Man Shing Wong* and Hung-Wing Li*

Department of Chemistry, Hong Kong Baptist University, Kowloon Tong, China

*To whom correspondence should be addressed. Email: mswong@hkbu.edu.hk, hwli@hkbu.edu.hk

Synthesis of the turn-on fluorophore

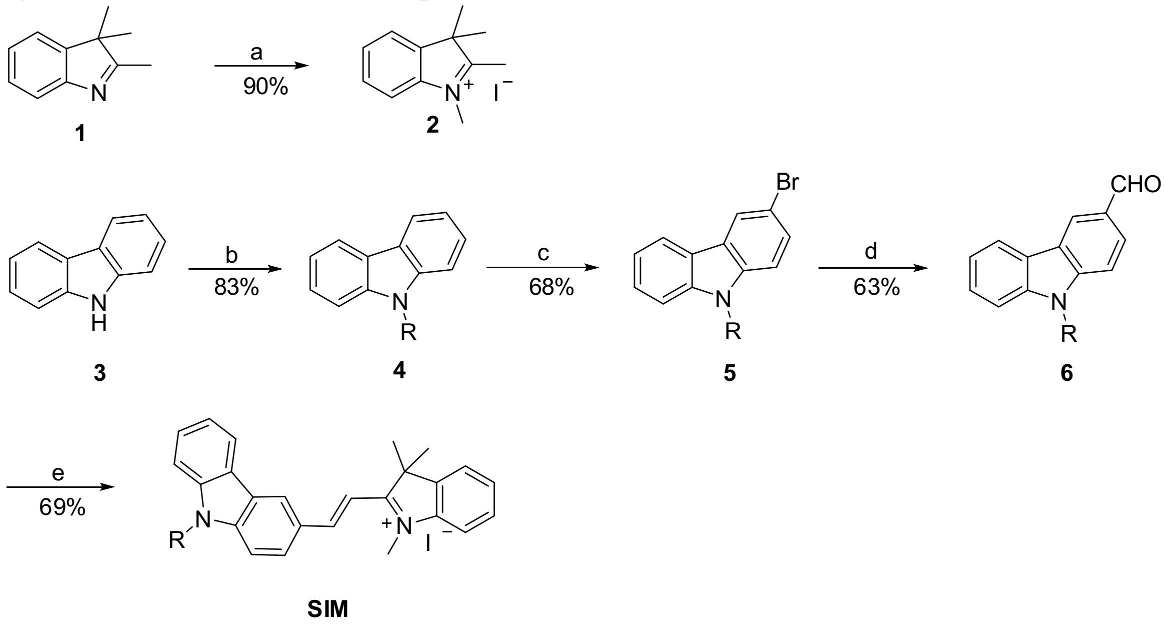

R = $CH_2CH_2OCH_2CH_2OCH_3$

Reagents and Conditions: a, CH_3I, sealed tube; b, $ClCH_2CH_2OCH_2CH_2OCH_3$, NaH, DMF, 75 °C; c, NBS, DCM, 0 °C to r.t.; d, (1) *n*-BuLi, THF, -78 °C (2) DMF; e, **2**, EtOH, reflux.

Scheme S1. Synthetic route of indolium-derived cyanine dyes, **SIM**.

General Procedure All the solvents were dried by the standard methods whenever needed. ^{1}H NMR spectra were recorded using a Bruker-400 NMR spectrometer and referenced to the residue $CDCl_3$ 7.26 ppm or DMSO-d_6 2.5 ppm. ^{13}C NMR spectra were recorded using a Bruker-400 NMR spectrometer and reference to the $CDCl_3$ 77 ppm or DMSO-d_6 39.5 ppm. Mass Spectroscopy (MS) measurements were carried out by using either fast atom bombardment on the API ASTER Pulser I Hybrid Mass Spectrometer or matrix-assisted laser desorption ionization-time-of-flight (MALDI-TOF) technique.

(*E*)-2-(2-(9-(2-(2-methoxyethoxy)ethyl)-9H-carbazol-3-yl)vinyl)-1,3,3-trimethyl-3H-indol-1-ium (SIM) ^{1}H NMR (400 MHz, DMSO-d_6) δ 9.12 (s, 1H), 8.64 (d, J = 16.0 Hz, 1H), 8.36 (d, J = 8.5 Hz, 1H), 8.26 (d, J = 7.6 Hz, 1H), 7.87-7.82 (m, 3H), 7.75-7.72 (m, 2H), 7.64-7.53 (m, 3H), 7.38-7.34 (m, 1H), 4.68-4.65 (m, 2H), 4.16 (s, 3H), 3.85-3.83 (m, 2H), 3.48-3.45 (m, 2H), 3.30-3.27 (m, 2H), 3.09 (s, 3H), 1.85 (s, 6H). ^{13}C NMR (100 MHz, DMSO-d_6) δ 181.1, 155.1, 143.9, 143.2, 141.9, 141.1, 128.9, 128.6, 126.8, 125.8, 124.8, 123.1, 122.8, 122.3, 120.6, 114.5, 110.9, 110.8, 109.3, 71.3, 69.8, 68.8, 58.1, 51.7, 43.1, 34.1, 25.8. HRMS (MALDI-TOF) m/z Calcd for $C_{30}H_{33}N_2O_2$ 453.2537 Found 453.2544[M^+].

Materials and Reagents. A Phosphate-buffered saline (PBS) was prepared by dissolving sodium phosphate monobasic dihydrate (Sigma), sodium phosphate dibasic heptahydrate (Sigma) and sodium chloride (Sigma) in distilled water. The pH of the buffer was adjusted to pH 7.4 with 2 M HCl. The buffer solution was filtered through a 0.22 μm nylon membrane filter.
The commercial available antibodies were purchased and used without further purification: 12F4 (SIG-39142, Covance), 4G8 (SIG-39220, Covance), 11A50-B10 (SIG-39140, Covance), HT7 (MN1000, Thermo Scientific), BT2 (MN1010, Thermo Scientific) and AT270 (MN1050, Thermo Scientific). The human CSF is purchased from PrecisionMed (US).
Human serum sample was purchased from GeneMay, CSF samples were purchased from PrecisionMed. Saliva and urine samples were obtained from healthy donors, who gave their consent for this study.

Pretreatment of coverslides. All the coverslides were prewashed prior to use. In short, No. 1 22 mm square glass slides (Gold Seal, Electron Microscopy System) were consecutively sonicated in household detergent for 10 min, distilled water for 10 min twice, acetone for 10 min and ethanol for 10 min. The slides were then soaked in Piranha solution (H_2SO_4:H_2O_2 3:1) for 30 min and sonicated for 30 min; then sonicated again in the solution of HCl:H_2O:H_2O_2 (1:1:1) at 60°C for 30min, further soaked in Piranha solution for 30 min, followed by sonication for 30 min. In between each step, all the slides were rinsed with filtered H_2O thoroughly. The slides were stored in filtered water and blow-dried with nitrogen gas before use.

Preparation of flow cell. The flow cell was prepared by combining the pretreated coverslides and the lower 20 × 32 mm coverslides with double-sided adhesive tapes with a channel width of approximated 3 mm each. The volume of each channel was about 6.6 μL.

Preparation of the silica-coated iron oxide nanoparticles
The iron oxide nanoparticles were prepared by co-precipitation of ferrous and ferric ion solutions in 1:2 molar ratios. The solution of 2.5 mM $FeCl_2$•$4H_2O$ and 5 mM $FeCl_3$•$6H_2O$ in 2 M HCl was added to 62.5 mL 0.7 M NH_4OH under mechanical stirring. Stirring was allowed for 30 min. The nanoparticles were washed and redispersed in 12.5 mL distilled water, followed by the addition of three aliquots of 2.5 mL 1M tetramethylammonium hydroxide solution under mechanical stirring for 24 h. The nanoparticles were washed as follows: 800 μL nanoparticles solution was diluted with 4 μL 2M HCl, centrifuged and redispersed in distilled water.
The silica coating was done by sol-gel reaction. An ethanolic solution of TEOS (98%, Aldrich) was added to a mixture of 4.85 mL NH_4OH, 28.8 mL distilled water, 27.5 mL EtOH and 1.6 mL of the previously washed magnetic nanoparticles under mechanical stirring. Stirring was allowed for 4 h. The nanoparticles were washed with ethanol and distilled water respectively, and then redispersed in 5 mL distilled water.

Preparation of the iron oxide nanoprobes. A solution of 100 μL silica coated iron oxide nanoparticles were added to an ethanolic solution of (3-aminopropyl)triethoxysilane (Aldrich) under stirring at 70 °C for 24 h. The resulting nanoparticles were further functionalized by glutaraldehyde (70%, Aldrich) under stirring for 2 h. In between each step, the nanoparticles were washed twice and redispersed in PBS. The prepared nanoparticles were incubated with capture antibodies (11A50-B10 for $A\beta_{40}$, 12F4 for $A\beta_{42}$, BT2 for tau_{441} and AT270 for p-tau_{181}) for 2 h. The resulting nanoparticles were washed twice and redispersed in PBS.

Optimization of the immunoassay conditions. To optimize the concentration of the turn-on fluorophore used for labelling, the immunocomplex were incubated with 1, 10, 20, 50, 100 and 200 μM dye. To determine the concentration of the detection probes that appropriate for the detection, 3 mg/mL, 600 μg/mL, 300 μg/mL and 30 μg/mL nanoprobes were used for the detection. To optimize the detection procedure, the co-incubation of the nanoprobes, target analyte and detection antibody and the separate incubation; with and without an extra washing step after the magnetic separation step were compared. To decide the concentration of the capture antibody for immunoassay, 100, 500 and 1000 pM capture antibody were added for the coupling with nanoparticles. To optimize the detection procedure, the co-incubation of the nanoprobes, target analytes and detection antibody and the separate incubation; with and without extra washing step after the magnetic separation were compared. To ascertain the reaction time for different step, the nanoprobes were incubated at 37 °C with the target analyte for 15, 30 and 60 min; the immunocomplex were incubated at 37 °C with the detection antibody for 15, 30, and 60 min. To study the matrix effect of the cerebrospinal fluid (CSF), the analyte was added into artificial CSF (aCSF, R&D Systems). To investigate whether excessive antibody places any significant were added for the immunoreaction.

Selectivity of the nanoprobes. To study the selectivity of the detection assay, four samples were prepared, probes for the detection of $A\beta_{42}$ incubated with (i) 0 fM beta amyloid proteins, (ii) 500 fM $A\beta_{40}$, (iii) 500 fM $A\beta_{42}$ and (iv) mixture of the beta amyloid proteins with final concentration of 500 fM each at the optimal condition. To study the specificity of the antibody, four channels immobilized with (i) 50 μM $A\beta_{40}$, (ii) 50 μM $A\beta_{40}$ with 500 nM $A\beta_{42}$ antibody (iii) 50 μM $A\beta_{42}$ and (iv) 50 μM $A\beta_{42}$ with 500 nM $A\beta_{42}$ were labelled with 500 μM SIM. The fluorescent images were captured by TIRFM-EMCCD system.

Detection of target protein biomarkers. The calibration curve was established by correlating the average net intensity of fluorescent molecules in captured images at each concentration of spiked target analyte. The net intensity can be calculated by subtracting the intensity of 1×1 square pixel of the magnetic immunocomplexes from that of individual background area on the image. The average net intensity was obtained by taking average of the net intensities of 100 individual magnetic immunocomplexes. Tau_{441}, phosphorylated tau at Thr181, 1,1,1,3,3,3-hexafluoro-2-propanol (HFIP, Aldrich) treated $A\beta_{40}$ and $A\beta_{42}$ monomer with a final concentration of 0, 5, 10, 50, 100, 250, 500 or 1000 fM was incubated with the nanoprobes and detection antibody at the optimal condition, followed by the labeling of the dye. 10 μL of the dye labeled immune-solution was injected into the flow cell followed by the magnetic separation.

Quantification of the target protein biomarkers in human serum. The standard addition curve and external calibration curve were established by correlating the average net intensity of fluorescent molecules in captured images at each concentration of spiked target analyte and target analyte respectively. HFIP treated $A\beta_{42}$ monomer with a final concentration of 0, 250, 500, 750, 1000, 1500, 2500, and 5000 fM was incubated with the 3x nanoprobes, serum, and detection antibody at the optimal condition, the excessive reagent was discarded after the immunoreaction by the magnetic separation. The nanoprobes were then redispersed in PBS and labeled with the dye. The dye labeled immune solution of 10 μL was injected into the flow cell.

Pretreatment of human saliva and urine sample. Both of the saliva and urine samples were pre-treated prior use. The saliva sample was centrifuged at 1500 rpm at 4 °C for 5 min to remove debris. The sample was then stored at –20 °C. For the urine sample, it was placed

in ice bath for 30 min. The urine sample was centrifuged at 13,500 rpm at 4 °C for 15 min. The supernatant of the sample was treated with 150 μL 100% (w/v) trichloroacetic acid, and left on ice for 1 h. The sample was centrifuged again at 13,500 rpm at 4 °C for 15 min. The pellets were washed with acetone for three times. The pellets were left to air-dry and stored at -20°C. The pellets were redispersed in PBS prior use.

Quantification of the target protein biomarkers in human CSF, urine and saliva. The external calibration curve was established by correlating the average net intensity of fluorescent magnetic immunocomplexes in captured images at each concentration of target analyte. Three independent calibrations for $A\beta_{42}$ monomer, tau_{441} and p-tau_{181} were established. In short, the target antigen of concentration ranges from 0-5 fM ($A\beta_{42}$) and 0-2.5 fM (tau_{441} and p-tau_{181}) was incubated with the nanoprobesand detection antibody at the optimal condition and the magnetic immunocomplexes were labeled with the dye. The dye labeled immune solution of 10 μL was injected into the flow cell. The magnetic separation was performed prior the imaging by the TIRFM. The CSF sample was diluted with PBS before the immunoassay. The detection assay for CSF, saliva and urine samples were performed using the same method as the standards. The fluorescence images were captured by TIRFM with an excitation wavelength 488 nm.

Verification of the developed assay with INNOTEST ELISA kits. INNOTEST β-Amyloid1-42, Phospho-tau(181P) and hTau Ag were purchased from Fujirebio (Belgium). The detection of $A\beta_{42}$, t-tau and p-tau_{181} was performed in duplicate following the manufacturer's assay protocol. For the quantification of $A\beta_{42}$, 25 μL of $A\beta_{42}$ standards and CSF sample were added into the capture antibody coated micro-wells followed by 75 μL biotinylated detection antibody. The mixtures were then incubated at room temperature for 1 hr. The wells were emptied and washed with 1x wash solution for five times. Then, 100 μL peroxidase-labeled streptavidin was added to the wells and incubated at room temperature for 30 min. The wells were emptied and washed with 1x wash solution for five times. Next, 100 μL of the TMB substrate was added to the wells and incubated in the dark at room temperature for 30 min. Finally, 50 μL of stop solution was added into each well and the plate was shaken carefully for 1 min. The absorbance at 450 nm was recorded by Benchmark Plus Microplate Reader. The quantification of t-tau was done as follows: 25 μL of t-tau standards and CSF sample were added into the capture antibody coated micro-wells followed by 75 μL biotinylated detection antibody. The mixtures were then incubated at room temperature for 16 hr. The wells were emptied and washed with 1x wash solution for five times. Then, 100 μL peroxidase-labeled streptavidin was added to the wells and incubated at room temperature for 30 min. The wells were emptied and washed with 1x wash solution for five times. Next, 100 μL of the TMB substrate was added to the wells and incubated in the dark at room temperature for 30 min. Finally, 50 μL of stop solution was added into each well and the plate was shaken carefully for 1 min. The absorbance at 450 nm was recorded by Benchmark Plus Microplate Reader. The level of p-tau was determined by following approaches. In short, 75 μL of t-tau standards and CSF sample were added into the capture antibody coated micro-wells followed by 25 μL biotinylated detection antibody. The mixtures were then incubated at 4°C for 16 hr. The wells were emptied and washed with 1x wash solution for five times. Then, 100 μL peroxidase-labeled streptavidin was added to the wells and incubated at room temperature for 30 min. The wells were emptied and washed with 1x wash solution for five times. Next, 100 μL of the TMB substrate was added to the wells and incubated in the dark at room temperature for 30 min. Finally, 50 μL of stop solution was added into each well and the plate was shaken carefully for 1 min. The absorbance at 450 nm was recorded by Benchmark Plus Microplate Reader.

Quantification of target protein biomarkers with spectrofluorimeter. To explore the possibility of the quantification of the biomarkers with commercial fluorimeter, the external calibration curve of $A\beta_{42}$ was established by correlating the fluorescent intensity at the emission maximum of the fluorophores against different concentrations of target proteins. Different concentrations of $A\beta_{42}$ (0-1000 fM) was incubated with the optimal amount of nanoprobes and detection antibody under the optimal condition in 10% glycerol-PBS solution at 37 °C for 1 h. The resultant immunocomplexes were labeled with 50 μM SIM and the fluorescence spectra of the immunocomplexes was recorded by the fluorescence spectrophotometer (PTI QM-4/2005). The quantification of $A\beta_{42}$ in human CSF was performed using the same method as the standards and the fluorescent signal was measured by the spectrophotometer.

Multiplex detection of target protein biomarkers. In order to demonstrate the multiplexity of the detection assay, 5 pM of the target protein, $A\beta_{42}$ and tau_{441}, were incubated with their corresponding probes labeled with two different fluorescent dye, **SLAce** and **SIM**, and detection antibody. The solution mixture was then incubated with SIM and injected into the flow cell. The first-order images were then observed under the TIRFM-EMCCD imaging system with a transmission grating.

Stability of the turn-on fluorescent dye, SIM. The photostability of the fluorescent dye was studied by measuring the fluorescent intensity of the solution with 1 mM fluorescent dye under the present and absent of $A\beta_{42}$ in 1x PB (50 mM PB, pH 7.4) for every 5 mins. To examine the influence of salt on the performance of the fluorescent dye, the 1 mM fluorescent dye was spiked in 1x PB with 0, 100, 200, 300, 400 and 500 mM NaCl with and without 100 nM $A\beta_{42}$. To investigate the influence from magnetic nanoparticles, the mixture of 1 mM dye and 100 nM $A\beta_{42}$ was spiked in 1x PB with and without 1 mg/mL magnetic nanoparticles. The fluorescent spectra were measured by the fluorescence spectrophotometer (PTI QM-4/2005).

	Solvent	λ^{abs}_{max}/nm	λ^{em}_{max}/nm[a]	Φ_{PL}
**SLAce**	PB	478	681	0.003[b]
**SIM**	PB	475	597	0.13[b]

[a]excited at the absorption maxima; [b] using Rhodamine 6G (Φ_{488} = 0.95) as standard.

Table S1. Summary of the physical properties of the cyanine fluorophores.

Saliva

Individual 1	ELISA (pg/mL)	% Difference	MICs (pg/mL)	RSD (%)	RPD (%)
$A\beta_{42}$	90.25	6.99	97.25	7.58	7.75
tau_{441}	56.65	0.07	61.77	3.08	9.03
p-tau_{181}	25.35	0.19	26.68	6.70	5.26

Individual 2	ELISA (pg/mL)	% Difference	MICs (pg/mL)	RSD (%)	RPD (%)
$A\beta_{42}$	113.68	7.02	108.11	2.08	-4.90
tau_{441}	443.85	11.76	457.27	9.77	3.02
p-tau_{181}	41.4	5.56	38.09	1.59	-7.99

Individual 3	ELISA (pg/mL)	% Difference	MICs (pg/mL)	RSD (%)	RPD (%)
$A\beta_{42}$	115.07	1.71	121.38	10.30	5.49
tau_{441}	276.48	7.69	294.11	6.45	6.38
p-tau_{181}	12.16	15.65	11.91	2.02	-2.07

Individual 4	ELISA (pg/mL)	% Difference	MICs (pg/mL)	RSD (%)	RPD (%)
$A\beta_{42}$	111.37	5.50	121.12	3.32	8.76
tau_{441}	329.63	2.44	306.60	1.51	-6.99
p-tau_{181}	13.27	3.45	13.93	1.39	4.95

Urine

Sample	ELISA (pg/mL)	% Difference	MICs (pg/mL)	RSD (%)	RPD (%)
$A\beta_{42}$	88.66	8.57	91.46	17.68	3.15
tau_{441}	107.30	0.15	112.67	4.94	5.01
p-tau_{181}	16.22	0.05	16.76	12.40	3.35

Table S2. Concentration of 3 biomarkers in two types of biological fluid, saliva and urine. (% difference = difference between duplicate / sum of duplicate / 2 x 100%; RSD (%) = SD / Mean x 100%; RPD (%) = difference obtained by two methods / concentration obtained by ELISA x 100%)

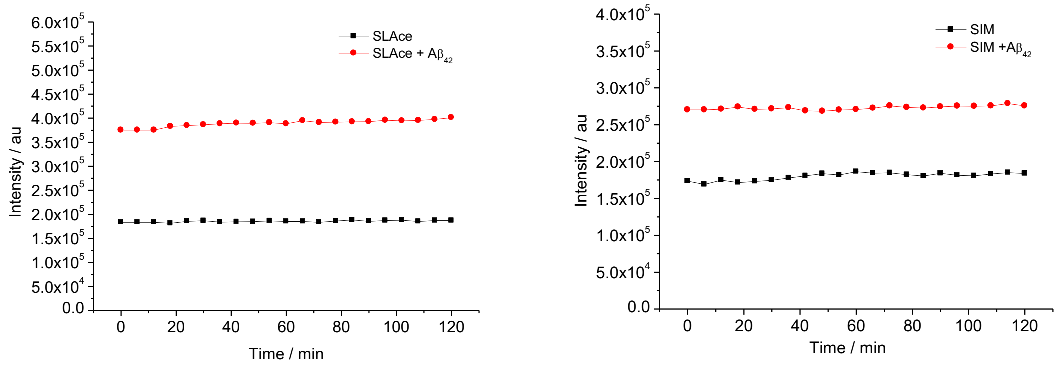

Figure S1. Photostability study of **SLAce** and **SIM** before and after binding to $A\beta_{42}$ in PB.

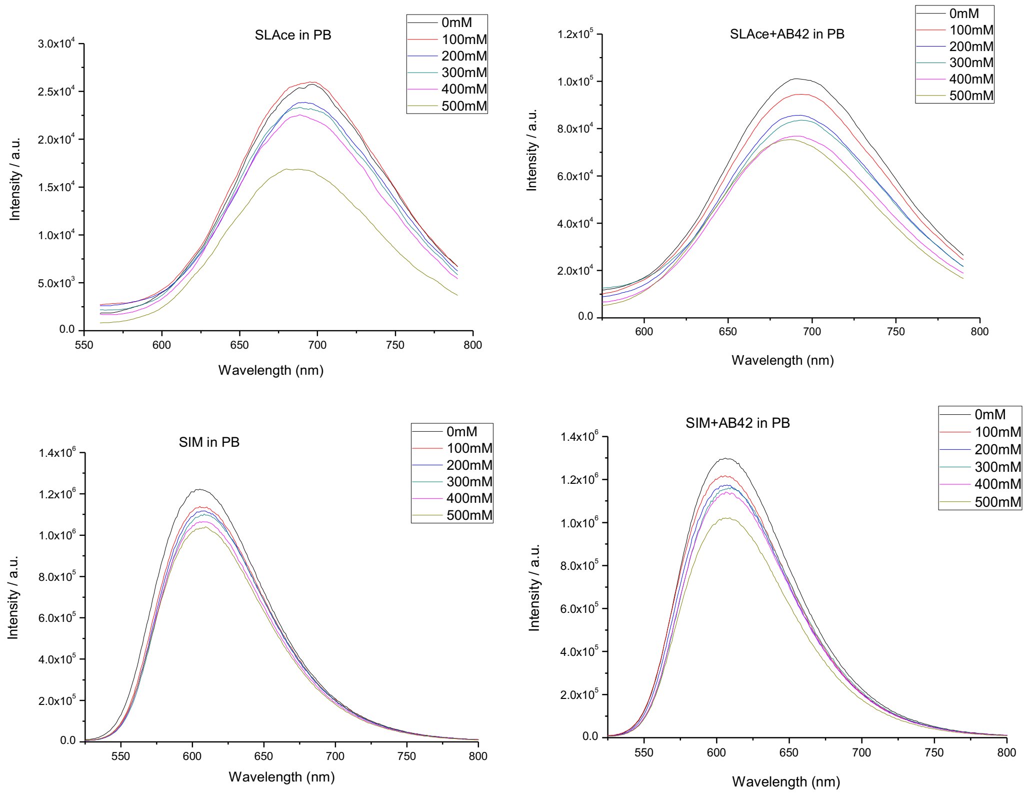

Figure S2. The fluorescence response of **SLAce** and **SIM** against salt concentration in the absence or presence of Aβ_{42} in PB buffer.

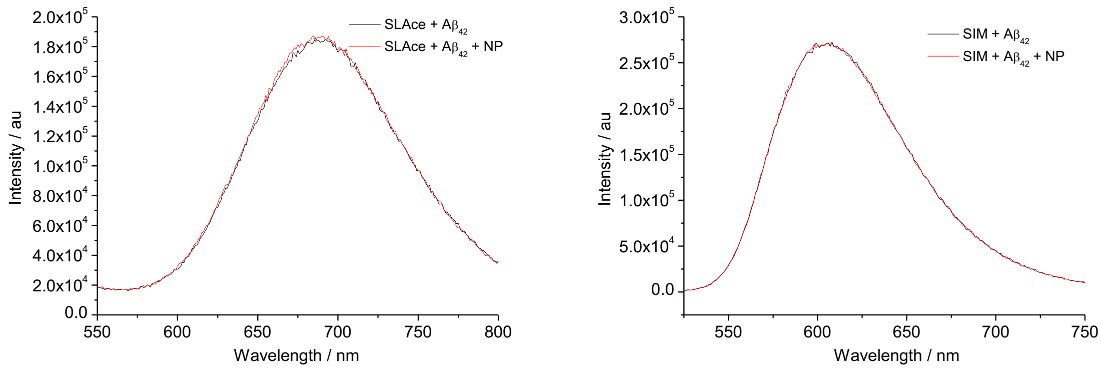

Figure S3. The influence of the magnetic nanoparticles on the fluorescence response of the **SLAce** and **SIM** upon binding to $A\beta_{42}$ in PB buffer.

A B

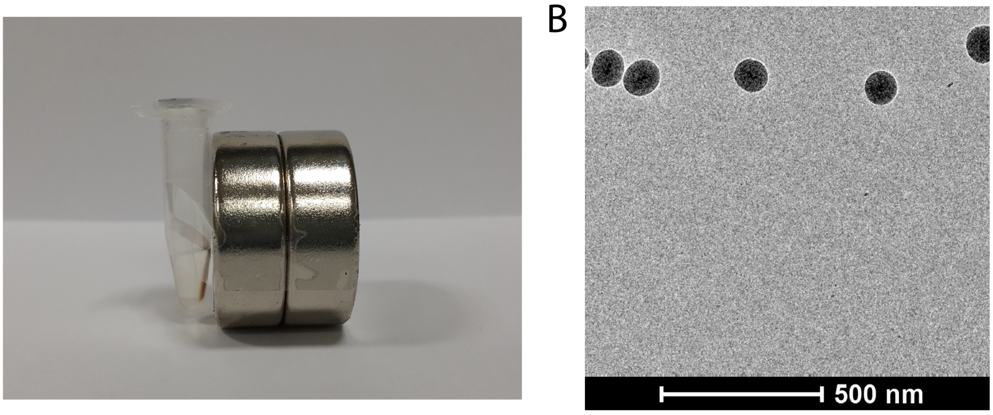

Figure S4. (A) A photo showing silica-coated iron oxide nanoparticles which originally disperse in aqueous solution in the presence of the external magnetic field. (B) A TEM image of the silica-coated iron oxide nanoparticles.

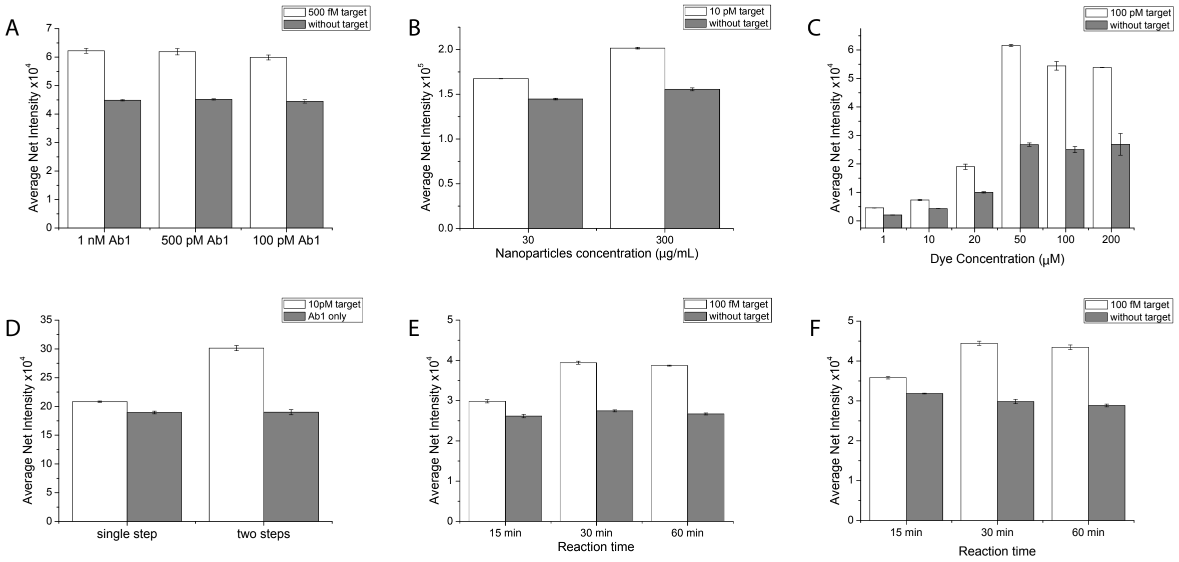

Figure S5. Optimization of (A) concentration of capture antibody, (B) concentration of capture antibody-conjugated nanoparticles, (C) concentration of dye, (D) incubation procedure , (E) reaction time for the immunoreaction between capture antibody conjugated nanoparticles with target protein (reaction 1) and (F) immunocomplexes formed by coupling the target with capture antibody and detection antibody (reaction 2). Error bars, standard error of mean n=3. (Average net intensity = (1×1 square pixel of 100 individual MICs) – (1×1 square pixel of 100 individual background area on the image)/100).

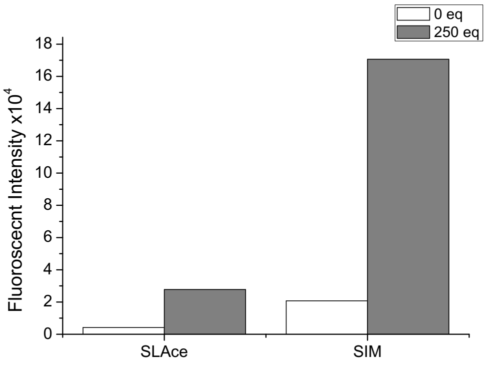

Figure S6. Fluorescent intensity of the mixture of 0 and 250 µM Aβ_{42} with same dye concentration of **SLAce** and **SIM**.

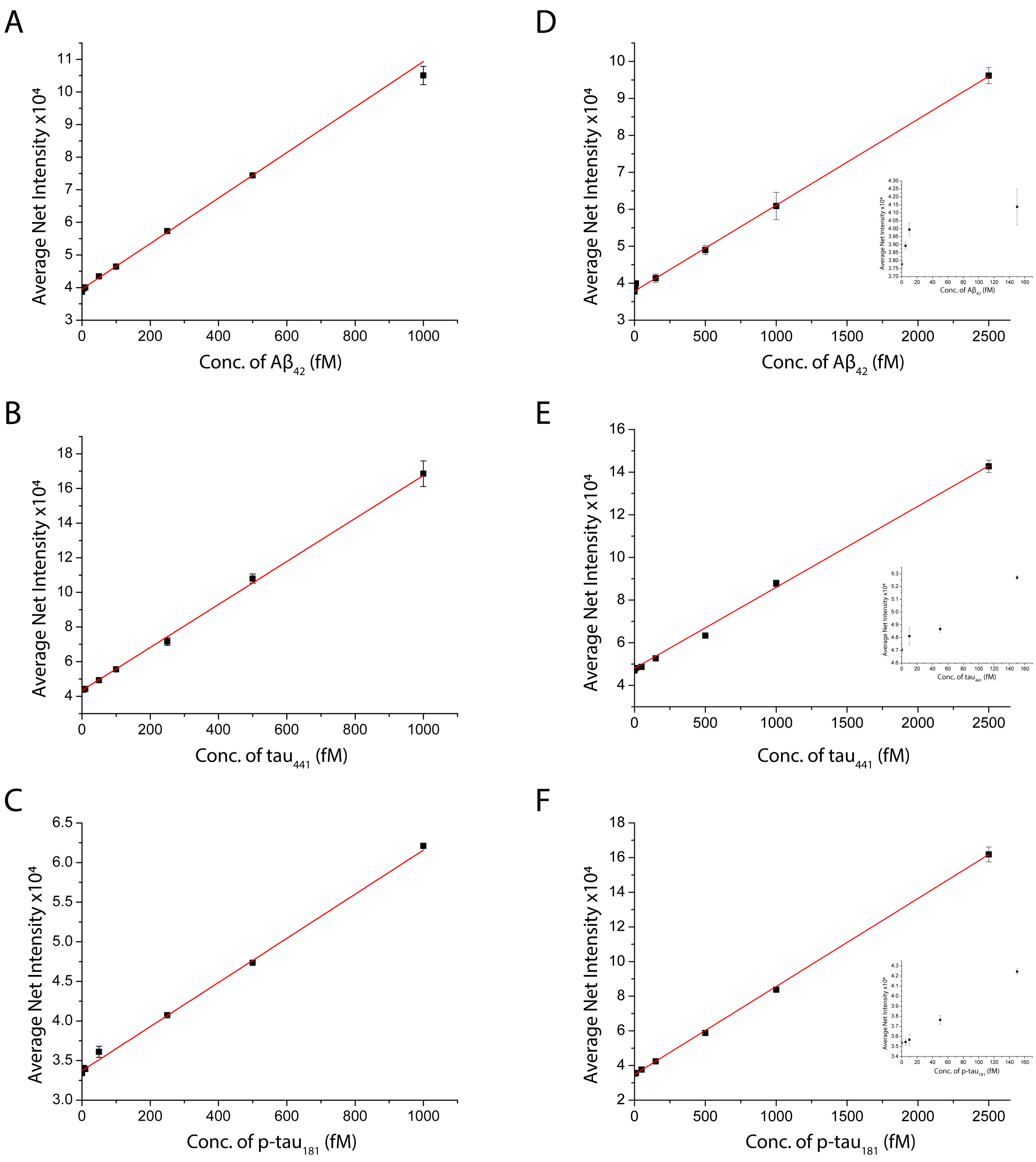

Figure S7. Calibration plot of the quantification of monomeric $A\beta_{42}$, tau_{441}, and p-tau_{181} by using (A-C) **SLAce** and (D-F) **SIM**. Different concentrations of target were incubated with the nanoprobes under optimal condition. Error bars, standard error of mean n=3. (Average net intensity = (1×1 square pixel of 100 individual MICs) – (1×1 square pixel of 100 individual background area on the image)/100). The limit of detection of $A\beta_{42}$, tau_{441}, and p-tau_{181} using **SLAce** as fluorescent dye are 50, 24 and 50 fM respectively, while that using **SIM** are 23, 14 and 34 fM respectively.

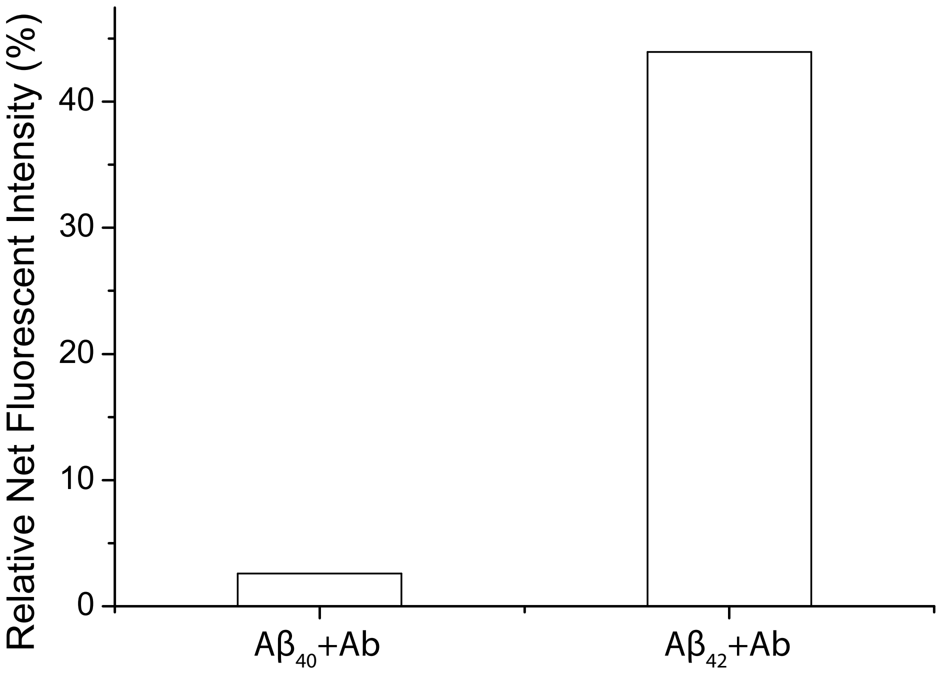

Figure S8. Specificity of the $Aβ_{42}$ antibody (clone 12F4). Relative Net Fluorescent Intensity = (Average of Integrated Density of 10 positions of Aβ proteins with 12F4 antibody – Average of Integrated Density of 10 positions of Aβ proteins only)/ Average of Integrated Density of 10 positions of Aβ proteins only ×100%.

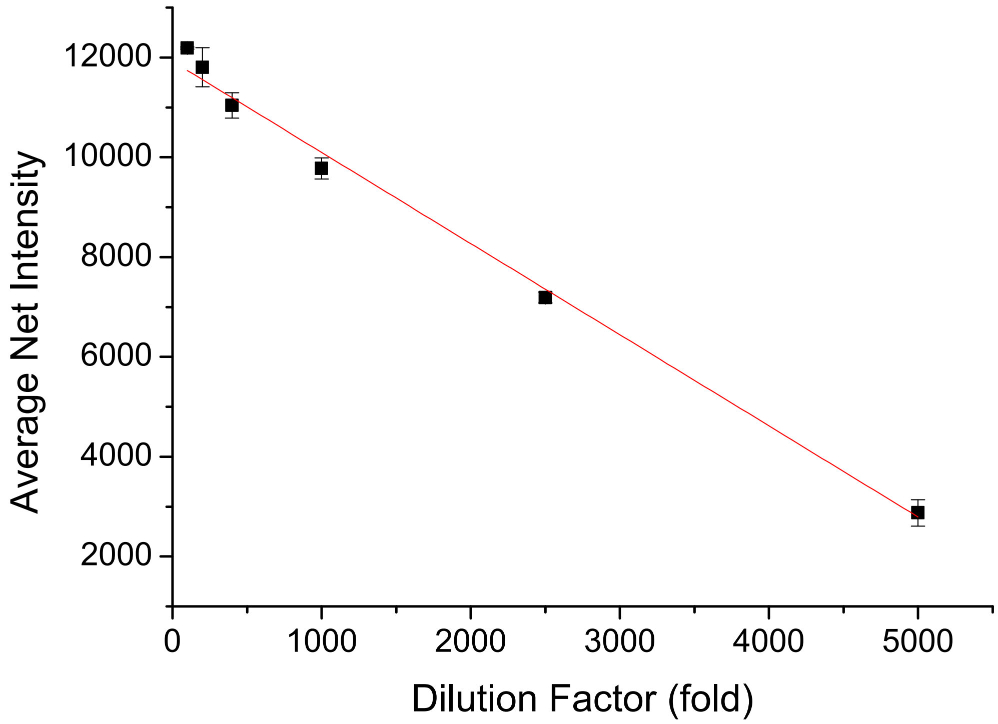

Figure S9. Dilution linearity of the Aβ_{42} in human CSF sample. Error bars, standard error of mean n=3. (Average net intensity = (1×1 square pixel of 100 individual MICs) – (1×1 square pixel of 100 individual background area on the image)/100).

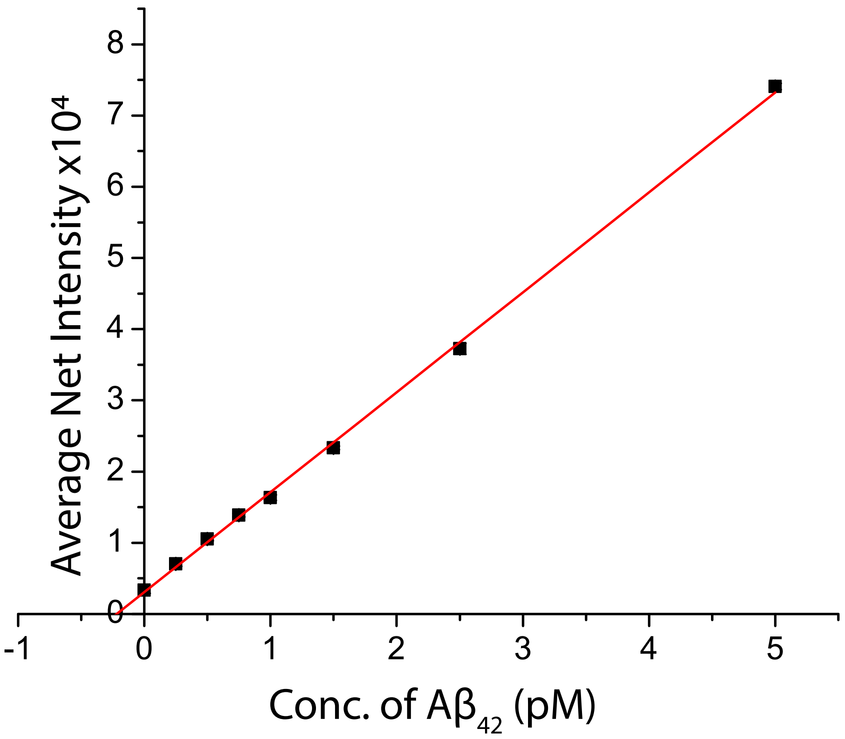

Figure S10. Quantification of the $A\beta_{42}$ in human serum sample by standard addition. Error bars, standard error of mean n=3. (Average net intensity = (1×1 square pixel of 100 individual MICs) – (1×1 square pixel of 100 individual background area on the image)/100). The concentration of $A\beta_{42}$ was 353.43 pg/mL.

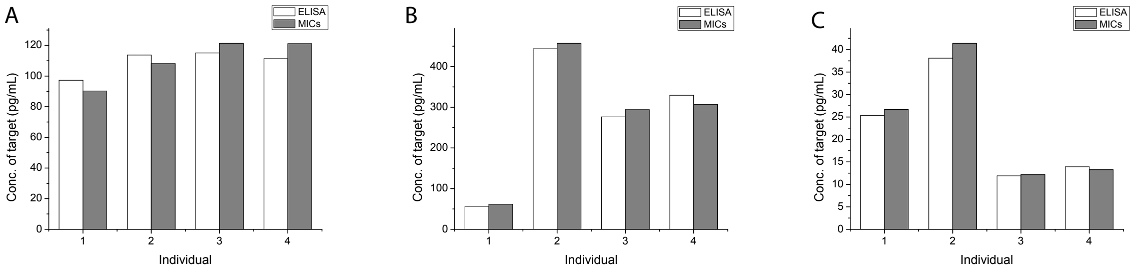

Figure S11. Quantification of (A) monomeric Aβ_{42}, (B) tau$_{441}$, and (C) p-tau$_{181}$ in saliva sample of four individuals by ELISA and the developed assay using SIM as reporter.

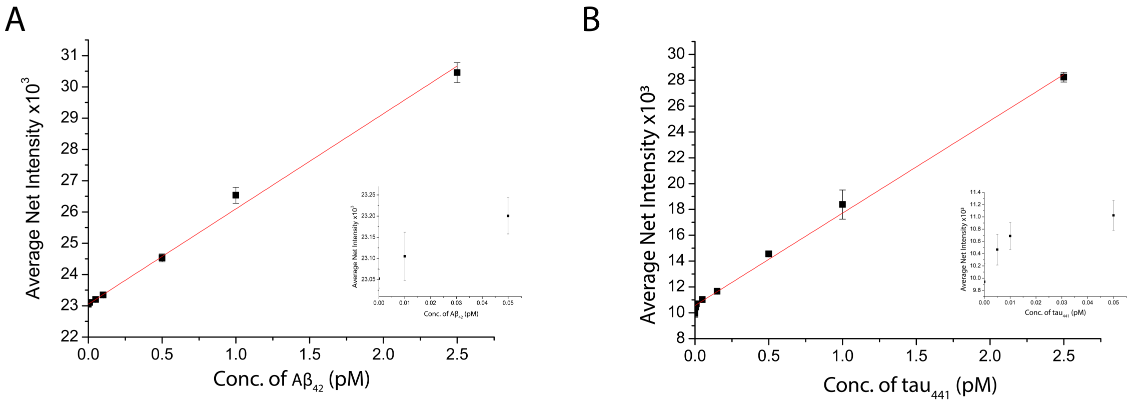

Figure S12. Calibration plot of the quantification of (A) $A\beta_{42}$ by using **SIM**-labelled probes and (B) tau_{441} by using **SLAce**-labelled probes. Different concentrations of target were incubated with the probes under the optimal condition. Error bars, standard error of mean n=3. (Average net intensity = (1×1 square pixel of 100 individual MICs) – (1×1 square pixel of 100 individual background area on the image)/100).
